# Case Report: Metastatic pancreatic ductal adenocarcinoma initially masked by multiple gastroduodenal ulcers with apparent endoscopic healing: a diagnostic pitfall

**DOI:** 10.3389/fmed.2026.1854821

**Published:** 2026-06-24

**Authors:** Conghua Song, Zhen Chen, Chaozhong Huang, Xiaomei Li

**Affiliations:** 1 Gastrointestinal Endoscopy Center, The Affiliated Hospital of Putian University, Putian, Fujian, China; 2 Gastrointestinal Endoscopy Center, Duwei Town Central Health Center, Xianyou, Fujian, China; 3 School of Basic Medicine, Putian University, Putian, Fujian, China; 4 Key Laboratory of Translational Tumor Medicine in Fujian, Putian University, Putian, Fujian, China

**Keywords:** carbohydrate antigen 19-9 (CA19-9), diagnostic errors, endoscopy, gastrointestinal, pancreatic ductal adenocarcinoma (PADC), peptic ulcer

## Abstract

**Background:**

Pancreatic ductal adenocarcinoma (PDAC) is often diagnosed at an advanced stage because its early manifestations are nonspecific and may mimic benign gastrointestinal disorders. When multifocal gastroduodenal ulcers show benign histology and apparent healing after acid-suppressive therapy, the diagnostic impression may be misleading and delay recognition of an underlying malignancy.

**Case presentation:**

A 69-year-old man presented with intermittent epigastric discomfort for 1 month. Upper gastrointestinal endoscopy revealed multiple ulcerative lesions involving the esophagus, stomach, and duodenum. Repeated biopsies showed only chronic inflammatory changes without dysplasia or malignancy. *Helicobacter pylori* testing was negative, and serum gastrin was within the normal range.

**Management and outcome:**

The patient was treated with high-dose vonoprazan (20 mg, bid, p.o.), and follow-up endoscopy demonstrated complete or near-complete healing of the ulcers. However, despite this favorable endoscopic response, the etiology of the extensive multifocal ulceration remained unexplained. Because this persistent diagnostic uncertainty was accompanied by ongoing symptoms and progressively rising carbohydrate antigen 19-9 (CA19-9) levels, further cross-sectional imaging was performed. Computed tomography and magnetic resonance imaging revealed a pancreatic tail mass with hepatic and peritoneal metastases. Laparoscopic biopsy of peritoneal nodules confirmed moderately differentiated adenocarcinoma, and immunohistochemical findings supported pancreatic ductal origin. A final diagnosis of metastatic PDAC was established.

**Conclusion:**

Apparent endoscopic healing of multifocal gastroduodenal ulcers does not exclude an underlying systemic malignancy. When the cause of extensive ulcerative disease remains unresolved, especially in the setting of persistent symptoms and dynamic CA19-9 elevation, timely pancreatic imaging should be considered to avoid delayed diagnosis of PDAC.

## Introduction

Pancreatic ductal adenocarcinoma (PDAC) is among the most lethal gastrointestinal malignancies and is frequently diagnosed at an advanced stage because its early manifestations are often nonspecific and easily attributed to more common benign gastrointestinal disorders (1). Delayed diagnosis remains a major clinical challenge, particularly when the initial presentation is dominated by symptoms or endoscopic findings that appear unrelated to pancreatic disease (2).

In routine gastroenterology practice, multifocal gastroduodenal ulceration with benign histopathological findings and favorable response to acid-suppressive therapy is generally interpreted as acid-related mucosal disease (3). However, when the etiology of extensive ulcerative lesions remains unclear, the diagnostic process should not be considered complete, even if follow-up endoscopy shows apparent mucosal healing. In such situations, alternative causes, including hypersecretory disorders such as *Zollinger-Ellison* syndrome (4), may need to be considered.

We report a case of metastatic PDAC initially masked by multiple gastroduodenal ulcers that showed apparent endoscopic healing after high-dose acid suppression. In this patient, the reason for further investigation was not solely persistent discomfort or rising carbohydrate antigen 19-9 (CA19-9), but also the unresolved question of why multifocal ulcerative disease had developed in the first place despite negative routine evaluation. Although hypersecretory conditions were considered, the eventual diagnosis was metastatic PDAC. This case highlights a clinically important diagnostic pitfall and underscores the need to integrate unexplained ulcer etiology, symptom evolution, laboratory dynamics, and cross-sectional imaging rather than relying solely on endoscopic resolution.

## Case description

A 69-year-old man presented with a 1-month history of intermittent epigastric discomfort without hematemesis, melena, vomiting, or dysphagia. He denied nonsteroidal anti-inflammatory drug use. His past medical history was unremarkable, with no history of diabetes mellitus, chronic pancreatitis, or previous peptic ulcer disease. He also denied significant weight loss, anorexia, smoking, alcohol misuse, and any known family history of gastrointestinal or pancreatic malignancy. Physical examination showed no jaundice, abdominal tenderness, palpable mass, clinically evident ascites, or superficial lymphadenopathy. The abdomen was soft and non-distended without shifting dullness.

Initial laboratory tests, including complete blood count, liver and renal function tests, serum electrolytes, and inflammatory markers, were within normal limits. Upper gastrointestinal endoscopy revealed multiple ulcerative lesions involving the duodenal bulb, gastric antrum, gastric angle extending to the lower gastric body, and the cardia. The ulcers were irregular and covered with fibrinous exudates, but no obvious mass-like protrusion or infiltrative lesion suggestive of primary upper gastrointestinal malignancy was identified (Figure 1). Concomitant reflux esophagitis was classified as Los Angeles grade C. Multiple biopsies were obtained from the ulcer margins and bases. Histopathological examination showed chronic inflammatory changes without dysplasia or malignant cells. *Helicobacter pylori* testing was negative by both rapid urease test and histological evaluation, and serum gastrin was within the normal range.

**Figure 1:**
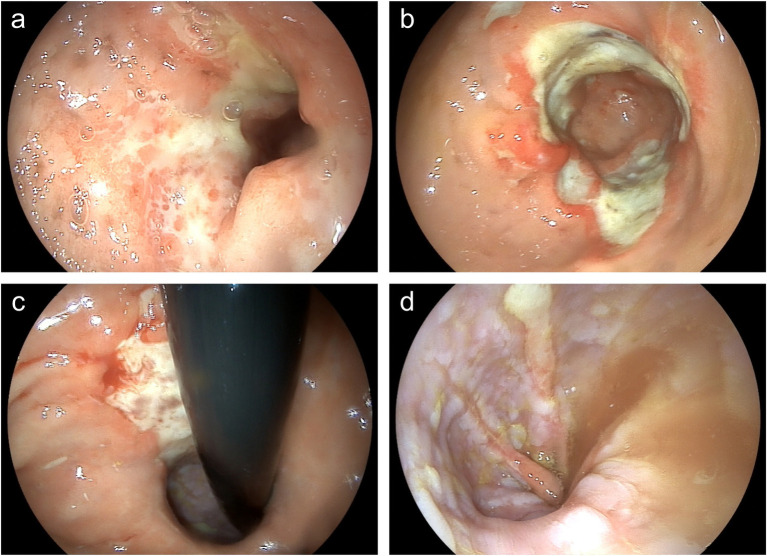
Endoscopic appearance of multifocal ulcerative lesions at initial presentation. **(a)** Ulcerative lesion in the duodenal bulb. **(b,c)** Irregular ulcerative lesions involving the gastric antrum and cardia, with fibrinous exudates and no obvious mass-like or infiltrative features. **(d)** Concomitant reflux esophagitis classified as Los Angeles grade C. Histopathological examination of biopsy specimens from the ulcer margins and bases showed chronic inflammatory changes without evidence of dysplasia or malignancy.

Based on the initial endoscopic and pathological findings, the patient was treated with high-dose vonoprazan (20 mg, bid, p.o.). Two weeks later, follow-up endoscopy demonstrated marked mucosal improvement, with complete or near-complete healing of the previously identified ulcers, now in the scar stage (Figure 2). Despite this favorable response to acid suppression, the underlying cause of the extensive multifocal gastroduodenal ulceration remained unexplained. Common causes of peptic ulcer disease, including *Helicobacter pylori* infection and nonsteroidal anti-inflammatory drug exposure, had been excluded, and overt hypergastrinemia was not present. Accordingly, the diagnostic process was not considered complete, and alternative etiologies, including hypersecretory disorders such as *Zollinger-Ellison* syndrome, remained under consideration.

**Figure 2:**
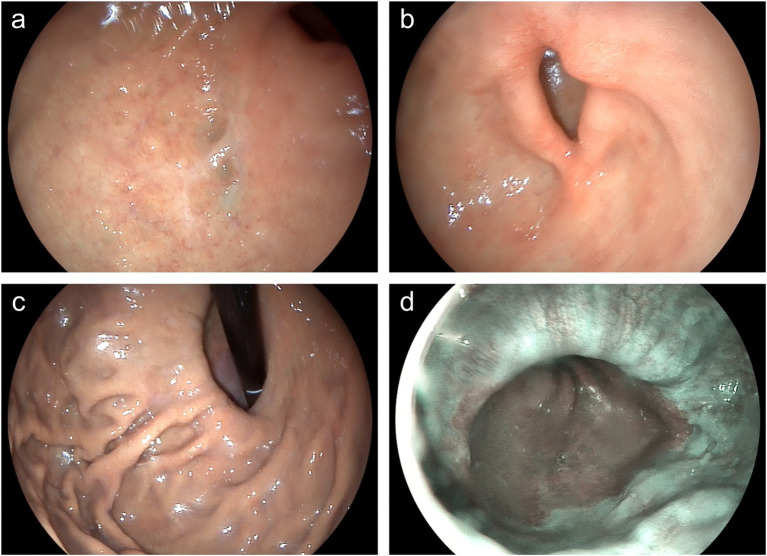
Follow-up endoscopic findings after high-dose vonoprazan therapy. **(a–c)** Previously identified ulcerative lesions showed complete or near-complete mucosal healing with scar formation. **(d)** Reflux esophagitis improved on follow-up examination. No residual ulcer crater or suspicious mass lesion was observed.

However, the patient continued to report persistent epigastric discomfort and dull upper abdominal pain despite apparent mucosal healing. Given the patient’s older age and the unresolved etiology of the multifocal ulcerative disease, evaluation beyond the gastrointestinal lumen was considered. Because the pancreas is anatomically adjacent to the stomach and may present with nonspecific upper abdominal symptoms, broader laboratory assessment including tumor marker screening (carcinoembryonic antigen, alpha-fetoprotein, and CA19-9) was performed. Serial testing demonstrated progressive elevation of CA19-9 from 82 U/mL to 436 U/mL over a 4-week interval. Repeat biochemical evaluation showed that complete blood count, liver function tests, including total bilirubin and alkaline phosphatase, and other routine laboratory parameters remained within normal limits, making cholestasis-related CA19-9 elevation less likely. Given the unresolved etiology of the multifocal ulcerative disease, the persistent symptoms, and the dynamic increase in CA19-9, further cross-sectional evaluation was undertaken.

Contrast-enhanced computed tomography and magnetic resonance imaging subsequently revealed a hypovascular mass in the pancreatic tail, accompanied by multiple hepatic and peritoneal lesions suspicious for metastatic disease (Figure 3). Endoscopic ultrasound-guided fine-needle aspiration was considered. However, because multiple metastatic deposits were readily accessible and histological confirmation was required, laparoscopic biopsy of peritoneal nodules was performed. Histopathological examination demonstrated moderately differentiated adenocarcinoma. Immunohistochemical staining showed positivity for CK7 and CK19, together with loss of SMAD4 expression, supporting a pancreatic ductal origin. On the basis of the clinical, radiologic, histopathologic, and immunophenotypic findings, a diagnosis of metastatic pancreatic ductal adenocarcinoma was established. No liver biopsy was performed because histopathological confirmation was obtained from laparoscopic biopsy of peritoneal nodules.

**Figure 3:**
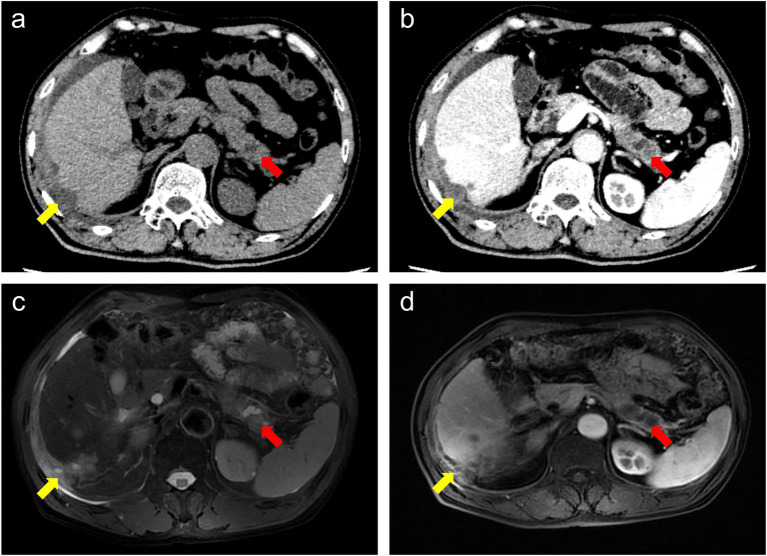
Cross-sectional imaging findings of metastatic pancreatic ductal adenocarcinoma. **(a,b)** Contrast-enhanced computed tomography demonstrating a hypovascular mass in the pancreatic tail and multiple hepatic and peritoneal lesions suspicious for metastatic involvement. **(c,d)** Magnetic resonance imaging demonstrating a heterogeneous pancreatic tail lesion with peripheral enhancement and multiple hepatic and peritoneal metastatic lesions. Red arrows (↑) indicate the primary pancreatic ductal adenocarcinoma, and yellow arrows (↑) indicate metastatic lesions.

## Timeline

To provide a clear overview of the diagnostic progression and clinical decision-making process, the major events in this case are summarized chronologically in a timeline table. This timeline highlights the evolution from initially unexplained multifocal gastroduodenal ulceration with apparent endoscopic healing to the eventual diagnosis of metastatic pancreatic ductal adenocarcinoma. The overall clinical course is summarized in Table 1.

**Table 1:** Timeline of clinical events.

Time point	Clinical findings	Investigations	Interpretation/action
Initial visit	Intermittent epigastric discomfort	Upper gastrointestinal endoscopy	Multiple upper gastrointestinal ulcerative lesions identified
Week 0	Multifocal gastroduodenal ulceration	Endoscopic biopsies, *Helicobacter pylori* testing, serum gastrin measurement	Common causes of peptic ulcer disease initially evaluated; high-dose acid suppression initiated
Week 2	Endoscopic improvement despite persistent symptoms	Follow-up upper gastrointestinal endoscopy	Marked mucosal healing with scar formation, but ulcer etiology remained unresolved
Week 4	Ongoing symptoms	Serial CA19-9 testing (82 U/mL to 436 U/mL)	Dynamic tumor marker elevation raised concern for an underlying extra-gastrointestinal process
Week 5	Persistent diagnostic uncertainty	Contrast-enhanced CT and MRI	Pancreatic tail mass with hepatic and peritoneal metastatic lesions detected
Week 6	Suspected metastatic pancreatic malignancy	Laparoscopic biopsy of peritoneal nodules	Histopathological confirmation of metastatic pancreatic ductal adenocarcinoma

## Diagnostic assessment

Initial diagnostic considerations included common causes of multifocal gastroduodenal ulceration, including *Helicobacter pylori* infection, nonsteroidal anti-inflammatory drug-related injury, hypersecretory conditions, inflammatory bowel disease, malignancy involving the upper gastrointestinal tract, and infectious etiologies. Repeated endoscopic biopsies demonstrated chronic inflammatory changes without dysplasia or malignancy. *Helicobacter pylori* testing was negative, and the patient denied nonsteroidal anti-inflammatory drug use. Clinical evaluation and laboratory findings provided no evidence supporting inflammatory bowel disease, systemic infection, or hypersecretory disorders.

Despite substantial endoscopic healing after proton pump inhibitor therapy, the patient continued to experience persistent epigastric discomfort and intermittent dull upper abdominal pain. Given the patient’s older age, unresolved multifocal ulcerative disease, and persistent symptoms despite apparent mucosal improvement, diagnostic evaluation was expanded beyond the gastrointestinal tract. Because pancreatic disorders may occasionally present with nonspecific upper abdominal symptoms and the pancreas is anatomically adjacent to the stomach and duodenum, additional laboratory assessment including tumor marker screening (CA19-9) was performed.

Serial testing demonstrated progressive elevation of CA19-9 from 82 U/mL to 436 U/mL over a 4-week interval, whereas bilirubin and cholestatic biochemical parameters remained within normal ranges. Contrast-enhanced computed tomography subsequently identified an ill-defined hypoenhancing lesion in the pancreatic tail with multiple hepatic lesions and peritoneal nodules suspicious for metastatic disease. Magnetic resonance imaging further supported metastatic pancreatic malignancy. Diagnostic laparoscopy with peritoneal biopsy was subsequently performed, and histopathological examination confirmed metastatic pancreatic ductal adenocarcinoma, establishing the final diagnosis.

## Therapeutic intervention

The patient was treated with vonoprazan, a potassium-competitive acid blocker (P-CAB), following identification of multifocal gastroduodenal ulcerative lesions. Follow-up endoscopy performed 2 weeks later demonstrated marked mucosal healing, indicating substantial endoscopic improvement after acid suppression therapy.

However, persistent epigastric discomfort and intermittent dull upper abdominal pain continued despite the apparent endoscopic improvement, prompting further diagnostic evaluation as described above. Following histopathological confirmation of metastatic PDAC, the patient was referred to the oncology service for multidisciplinary evaluation and subsequent management planning.

## Follow-up and outcomes

Follow-up endoscopy demonstrated marked healing of the multifocal gastroduodenal ulcerative lesions after vonoprazan treatment. However, persistent epigastric discomfort and intermittent dull upper abdominal pain despite apparent endoscopic improvement prompted further diagnostic evaluation, ultimately leading to identification of metastatic pancreatic ductal adenocarcinoma approximately 6 weeks after the initial presentation.

Following histopathological confirmation of the diagnosis, the patient was referred to the oncology service for subsequent management. At the time of manuscript preparation, systemic treatment had not yet been initiated. Comprehensive molecular profiling, including next-generation sequencing, had not been performed because the patient had transitioned to oncology-directed care outside the reporting clinical service.

## Discussion

Common presenting symptoms in PDAC cases, including epigastric discomfort, dyspepsia, nausea, bloating, and abdominal pain, frequently overlap with benign gastrointestinal disorders, contributing to delayed recognition and diagnosis (5, 6). In the present case, metastatic PDAC was ultimately diagnosed in a patient initially presenting with multifocal gastroduodenal ulcerative lesions and chronic upper abdominal discomfort, creating a complex diagnostic scenario.

Multifocal gastroduodenal ulceration has a broad differential diagnosis (3). Common etiologies include *Helicobacter pylori* infection and nonsteroidal anti-inflammatory drug–related injury, whereas less common causes include *Zollinger–Ellison* syndrome, Crohn’s disease, cytomegalovirus infection, vasculitis, infiltrative disorders, and malignancy-associated processes (7, 8). In this patient, repeated biopsies demonstrated only chronic inflammatory changes without dysplasia or malignancy, *Helicobacter pylori* testing was negative, and the patient denied nonsteroidal anti-inflammatory drug use. Clinical and laboratory evaluation also provided no evidence supporting inflammatory bowel disease, systemic infection, or hypersecretory disorders. Despite extensive evaluation, the underlying cause of the multifocal ulcerative lesions remained uncertain during the initial diagnostic stage.

Although a direct causal relationship between PDAC and the ulcerative lesions could not be established, malignancy may occasionally be associated with upper gastrointestinal ulceration through several mechanisms, including direct tumor invasion, metastatic involvement, local extension into adjacent structures, ischemic injury, or paraneoplastic effects (9). Previous reports have described gastric or duodenal ulcerative lesions secondary to pancreatic malignancy, but these cases generally involved histologically confirmed tumor infiltration or direct extension (10, 11). In contrast, no evidence of malignant involvement of the upper gastrointestinal mucosa was identified in our patient. Therefore, the ulcerative lesions are more appropriately interpreted as a coexisting and potentially misleading clinical finding rather than a confirmed manifestation of PDAC.

Several reports have also described pancreatic malignancy presenting initially with nonspecific upper gastrointestinal symptoms that were attributed to more common gastrointestinal disorders before pancreatic pathology was ultimately recognized (12, 13). These observations highlight the diagnostic challenges posed by PDAC and provide important clinical context for the present case. The significance of this report therefore lies not in proposing a novel biologic association between PDAC and multifocal ulcerative disease, but in illustrating how an initially plausible explanation for symptoms may inadvertently contribute to diagnostic anchoring and delayed recognition of an underlying malignancy.

An important question raised by this case is whether earlier suspicion of an extra-gastrointestinal process could have altered the clinical course. In our patient, follow-up endoscopy demonstrated marked healing of the gastroduodenal ulcers after vonoprazan therapy, which initially reduced concern for an ongoing serious underlying condition. However, persistent symptoms despite apparent endoscopic improvement, together with unresolved diagnostic uncertainty and progressive elevation of CA19-9 in the absence of cholestasis, ultimately prompted further investigation and led to the diagnosis of metastatic PDAC approximately 6 weeks after the initial presentation. Because metastatic disease was already present at diagnosis, it cannot be determined retrospectively whether earlier recognition would have altered long-term oncologic outcomes. Nevertheless, earlier consideration of pancreatic pathology in selected patients with unexplained clinical findings may potentially shorten diagnostic delay and facilitate earlier oncologic assessment and treatment planning (14).

This case also underscores a broader principle in clinical decision-making. Apparent improvement in local findings should not automatically terminate diagnostic reasoning when important etiologic questions remain unresolved. Persistent symptoms, discordance between treatment response and overall clinical evolution, or evolving objective abnormalities should prompt reconsideration of the diagnostic strategy. In selected symptomatic patients, dynamic elevation of CA19-9 without biochemical evidence of cholestasis may further support broader evaluation beyond the gastrointestinal tract (15, 16). Endoscopic ultrasound (EUS), because of its high sensitivity for small pancreatic lesions, may provide additional diagnostic value when conventional evaluation remains nondiagnostic and clinical suspicion persists.

At the same time, indiscriminate expansion of pancreatic investigation is neither practical nor cost-effective (17). Most patients with upper abdominal discomfort do not require advanced pancreatic imaging or EUS. Rather than advocating broader screening based on isolated findings, this report emphasizes risk-adapted escalation of diagnostic evaluation in selected patients with persistent symptoms, unresolved diagnostic uncertainty, discordance between apparent local improvement and overall clinical evolution, or evolving objective abnormalities. Earlier recognition of such diagnostic discordance may improve diagnostic timeliness while remaining mindful of healthcare resource allocation (18).

An important clinical implication of this case is that endoscopic improvement should not be equated with diagnostic closure. Acid suppression can promote mucosal healing even when the underlying pathophysiologic trigger has not been fully identified. In this patient, the visual resolution of ulcerative lesions could easily have reinforced an initial benign interpretation and led to premature reassurance. However, persistent epigastric discomfort after apparent healing, together with the unresolved etiology of multifocal ulceration, indicated that further evaluation remained necessary. In this context, the later rise in CA19-9 did not function as an isolated trigger, but rather as an additional warning signal that strengthened the need for cross-sectional imaging.

Several limitations should also be acknowledged. First, a causal relationship between PDAC and the multifocal ulcerative lesions cannot be established because no direct histopathologic evidence of malignant involvement of the upper gastrointestinal mucosa was identified. Second, serum amylase and lipase were not obtained during the initial evaluation because the patient presented with chronic rather than acute symptoms and had no clinical features or history suggestive of pancreatitis, which may have limited early characterization of pancreatic involvement. Third, comprehensive molecular profiling was unavailable because the patient transitioned to oncology-directed care before additional genomic characterization could be performed. Finally, systemic antitumor therapy had not yet been initiated at the time of manuscript preparation, limiting assessment of longer-term clinical outcomes.

## Patient perspective

Initially, the patient did not consider his symptoms to be serious. After the initial endoscopic findings and subsequent mucosal healing, he also tended to believe that the condition was related to peptic ulcer disease. However, as his symptoms persisted and the cause of the multifocal ulcerative lesions remained unclear, he understood the need for further evaluation for a possible extra-gastrointestinal disorder. After the final diagnosis of metastatic pancreatic ductal adenocarcinoma was established, the patient expressed regret about the severity of the disease but accepted the recommended oncologic evaluation and further treatment planning.

## Conclusion

In summary, this case underscores that apparent endoscopic healing of multifocal gastroduodenal ulcers does not necessarily indicate that the underlying disease process has been identified. When the cause of extensive ulcerative disease remains unexplained, especially if persistent symptoms and dynamic CA19-9 elevation coexist, further pancreatic imaging and continued diagnostic reassessment should be considered. Recognition of discordance between apparent endoscopic improvement and the overall clinical trajectory may help reduce anchoring bias, avoid premature diagnostic closure, and facilitate more timely evaluation of occult pancreatic disease. This case further emphasizes risk-adapted escalation of diagnostic evaluation in selected symptomatic patients with unresolved clinical uncertainty.

## Data Availability

The original contributions presented in the study are included in the article/supplementary material, further inquiries can be directed to the corresponding author.
